# How do cotton light interception and carbohydrate partitioning respond to cropping systems including monoculture, intercropping with wheat, and direct-seeding after wheat?

**DOI:** 10.1371/journal.pone.0217243

**Published:** 2019-05-20

**Authors:** Xiaoyu Zhi, Yingchun Han, Fangfang Xing, Yaping Lei, Guoping Wang, Lu Feng, Beifang Yang, Zhanbiao Wang, Xiaofei Li, Shiwu Xiong, Zhengyi Fan, Yabing Li

**Affiliations:** Institute of Cotton Research of the Chinese Academy of Agricultural Sciences/State Key Laboratory of Cotton Biology, Anyang, Henan, P. R. China; Murdoch University, AUSTRALIA

## Abstract

Different cotton (*Gossypium hirsutum* L.)-wheat (*Triticum aestivum*) planting patterns are widely applied in the Yellow River Valley of China, and crop yield mainly depends on light interception. However, little information is available on how cotton canopy light capturing and yield distribution are affected by planting patterns. Hence, field experiments were conducted in 2016 and 2017 to study the response of cotton canopy light interception, square and boll distribution, the leaf area index (LAI) and biomass accumulation to three planting patterns: a cotton monoculture (CM, planted on 15 May) system, a cotton/wheat relay intercropping (CWI, planted on 15 May) system, in which three rows of wheat rows were intercropped with one row of cotton, and a system in which cotton was directly seeded after wheat (CWD, planted on 15 June). The following results were obtained: 1) greater light capture capacity was observed for cotton plants in the CM and CWI compared with the CWD, and the light interception of the CM was 22.4% and 51.4% greater than that of the CWI and CWD, respectively, at 30 days after sowing (DAS) in 2016; 2) more bolls occurred at the first sympodial position (SP) than at other SPs for plants in the CM; 3) based on the LAI and biomass accumulation, the cotton growth rate was the greatest in CWD, followed by CM and CWI; and 4) the CM produced significantly greater yields than did the other two treatments because it yielded more bolls and greater boll weight. Information on the characteristics of cotton growth and development in response to different planting patterns would be helpful for understanding the response of cotton yields to planting patterns and would facilitate the improvement of cotton productivity.

## 1. Introduction

Cotton (*Gossypium hirsutum* L.) is grown worldwide as a major source of natural fiber [1]. In China, cotton is grown on 5.2 million hectares, and the annual production is 6.67 million tons [2]. Different cotton-wheat (*Triticum aestivum* L.) double cropping systems are commonly implemented in various countries worldwide, especially in the Yellow River Valley of China [3]. Double cropping systems are the collective planting of two or more crops within the same field, and compared with monoculture cropping systems, double cropping systems greatly contribute to crop production via the effective use of resources. Moreover, double cropping systems present several other beneficial attributes, such as high light interception [4], high productivity per unit area of land [5], efficient use of both water and nutrients, sequestration of organic carbon and nitrogen in the soil, and suppression of pests and diseases. Additionally, double cropping systems produce substantial increases in multiple crop indexes and reduce competition between grain and cotton for land in China. Further, cotton sown into mature wheat fields prior to or after wheat harvest improves the distribution of labor over time.

Moreover, double cropping systems are important factors that influence cotton growth and yield components [6]. Owing to the indeterminate growth habit of cotton plants, cotton crops display morphological adaptations to their growth environment, including modifications to their canopy structure in response to planting patterns [7–10]. Morphological adaptations with respect to canopy development, light interception, source-sink relationships and assimilate partitioning are the major determinants of lint yield [11–12]. Moreover, light interception is also highly influenced by different planting patterns [13], since the canopy structure changes in response to planting patterns, and the more efficient capture and use of light contribute to yield advantages of double cropping systems compared with monoculture systems [4, 14]. Therefore, the detailed study of cotton canopy light interception in response to different planting patterns allows a better understanding of how cotton canopy light interception and carbohydrate distribution are altered by planting patterns, but these phenomena are not yet clear.

Therefore, the objectives of the present study were to 1) calculate the variation of light interception due to different planting patterns, including a cotton monoculture (CM) system, a cotton/wheat relay intercropping (CWI) system and a system in which cotton is directly seeded after wheat (CWD); 2) quantify the effects of planting patterns on cotton fruit accumulation, shedding rate, leaf area index (LAI), and biomass accumulation; and 3) investigate differences in cotton yields.

## 2. Materials and methods

### 2.1. Experimental site

Field experiments were conducted in 2016 and 2017 at the research station of the Institute of Cotton Research, Chinese Academy of Agricultural Sciences, Anyang, Henan, China (36° 06' N, 114° 21' E). The same field was used both years and was characterized as having medium loam soil. The relevant soil characteristics prior to cotton sowing are listed in Table 1. During the cotton developmental stage, weather data were acquired from a weather station (Campbell Scientific, Logan, UT, USA) located near the experimental field (Table 2).

**Table 1:** Soil fertility of the experimental site in 2016 and 2017.

Year	Depth (cm)	Planting pattern	Organic matter (g kg^-1^)	Total nitrogen (g kg^-1^)	Available phosphorus (mg kg^-1^)	Available potassium (mg kg^-1^)
2016	0–20	CM	14.90	0.94	64.13	237.60
		CWI	15.55	1.08	65.35	235.57
		CWD	15.92	0.97	63.96	237.03
	20–40	CM	10.67	0.63	45.97	99.43
		CWI	11.53	0.83	47.66	104.63
		CWD	11.78	0.68	46.12	95.02
	40–60	CM	4.56	0.51	37.62	59.73
		CWI	5.86	0.51	37.90	62.98
		CWD	4.16	0.41	38.05	58.98
2017	0–20	CM	13.56	1.64	38.52	272.21
		CWI	14.04	2.06	36.01	260.03
		CWD	14.08	1.90	37.84	268.44
	20–40	CM	8.83	1.09	16.62	124.51
		CWI	8.17	1.34	14.91	120.59
		CWD	9.67	1.34	15.52	123.24
	40–60	CM	5.17	0.73	4.63	84.55
		CWI	6.44	0.93	5.11	82.85
		CWD	5.75	0.77	4.89	83.58

Note: CM, CWI, and CWD represent a cotton monoculture system, a cotton/wheat intercropping system, and a system in which cotton was directly seeded after wheat, respectively.

**Table 2:** Weather information during the cotton growing seasons in 2016 and 2017.

Meteorological variable	Daily mean temperature (°C)		Daily PAR (μmol s^-1^ m^-2^)		Precipitation (mm)		Sunshine duration (h)	
Year	2016	2017	2016	2017	2016	2017	2016	2017
Apr.	17.3	16.6	328.1	330.7	14.5	35.2	216.4	204.9
May	20.5	23.2	377.3	491.6	19.5	42.2	220.7	306.9
Jun.	26.2	25.8	442.3	435.4	70.9	63.8	234.5	240.7
Jul.	27.3	28.2	349.4	374.4	310.4	105.5	170.8	187.4
Aug.	26.3	26.5	376.3	357.7	52.7	28	192.9	191.7
Sep.	23	23	299.7	306.1	4.3	25.4	210	177
Oct.	15.6	14.3	165	174.3	46.9	25.6	102	97.7

### 2.2. Experimental design

The field experiments comprised three planting patterns: CM, CWI and CWD systems. The plots were arranged in a randomized complete block design, with three repetitions. The wheat variety ZY-36 and cotton variety YZ-9110 were used. Wheat was harvested on 10 June 2016 and 2017, and cotton for the CM and CWI systems was sown on 15 May 2016 and 2017. In addition, cotton for the CWD was sown on 15 June 2016 and 2017. Each individual plot area was 31.5 m^2^, with a length of 9.0 m and a width of 3.5 m, and each plot contained six rows of cotton.

**Fig 1:**
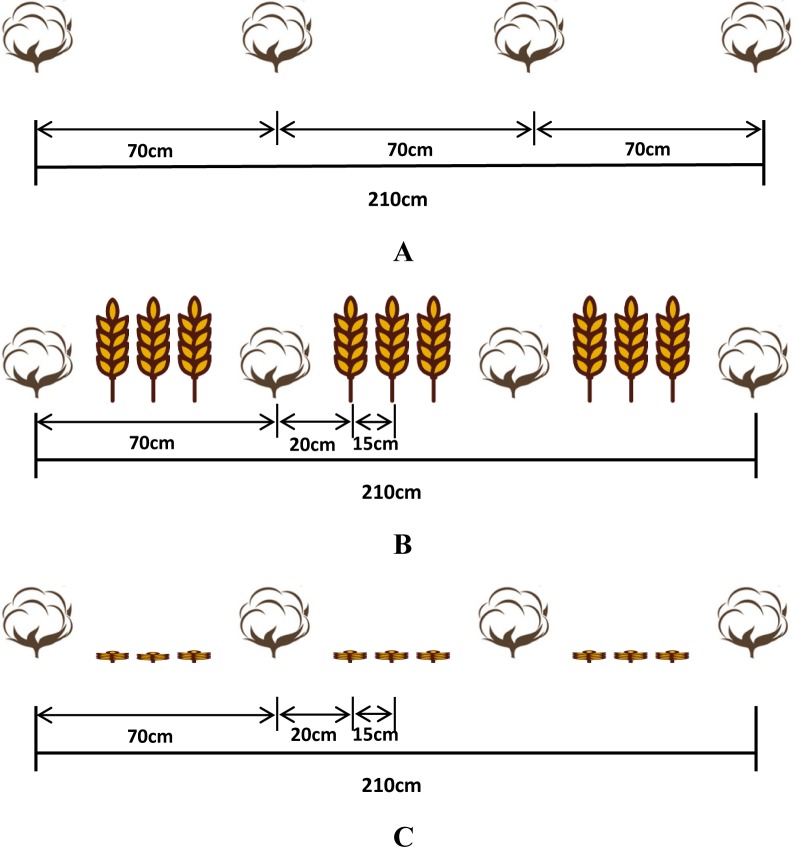
Layouts of the cotton monoculture (CM) system (A), the cotton/wheat intercropping (CWI) system (B), and the system in which cotton was directly seeded after wheat (CWD) (C).

The layout of the cropping systems is shown in Fig 1. Wheat was sown at an interrow distance of 15 cm, and cotton was sown at a density of 8000 plants ha^-1^, with an interrow distance of 70 cm. The cotton and wheat were sown at the same density across all treatments. In the CWI, winter wheat was sown in rows interspersed with bare soil left for cotton, and one row of cotton alternated between three rows of wheat. In the CWD, cotton was planted while wheat was harvested.

### 2.3. Field management

Agronomic management was consistent and based on local agronomic practices among all the treatments in 2016 and 2017. All fertilizers were broadcasted evenly across the soil and incorporated into the top 20 cm of the soil before sowing. During the wheat growth period, N, P, and K compound fertilizer (13:17:15) was applied as a starter fertilizer to supply 78 kg ha^-1^ N, 102 kg ha^-1^ P_2_O_5_ and 90 kg ha^-1^ K_2_O before wheat sowing. Furrow irrigation was applied at 600 m^3^ ha^-1^ on 16 March 2016 and 2017. After the cotton was planted, an additional 135 kg ha^-1^ N was applied during the cotton flowering stage. Irrigation was applied at a total volume of approximately 40 m^3^ by flooding the furrows in accordance with the recommendations of local agronomists. Weeds were manually controlled, and pesticides were used to control insects and disease.

### 2.4. Data collection

#### 2.4.1. Light interception

Instantaneous light capture by rows in the CM, CWI and CWD systems was determined throughout the whole growing season by measuring the light interception of photosynthetically active radiation (PAR) in the different planting patterns at 10-day intervals in 2016 and 2017. Both transmitted and reflected PAR in each layer of the canopy were measured in accordance with the spatial grid method [15], and the incident PAR above the canopy was measured at the same time. The transmitted and reflected PAR between 2 rows of cotton were measured with 100-cm quantum meters (LI-191SA, LI-COR, Lincoln, NE, USA) and data-loggers (LI-1400, LI-COR, Lincoln, NE, USA) under clear or partly cloudy conditions at 10:00 a.m. The quantum meters were oriented parallel to the crop rows and were fixed at different positions along a transect across the rows at intervals of 14 cm. In the vertical direction, each meter was moved from the ground to top of the plants at 20-cm intervals. The PAR data from two years were interpolated via kriging [16] and the use of Surfer 13 (Golden Software Inc., USA) to determine the light interception at different growth stages.

#### 2.4.2. Agronomic traits

Agronomic traits (plant mapping, LAI and biomass) were also measured at 10-day intervals throughout the whole growing season, beginning at 20 days after sowing (DAS) until harvest. To avoid any border effects, 10 cotton plants within the middle row of each plot were randomly sampled for plant mapping; the square fraction (the number of squares per specific node divided by the number of plants sampled), boll fraction (the number of bolls per specific node divided by the number of plants sampled), and shedding rate (the number of fruit-shedding sites per specific node divided by the number of plants sampled) were determined. Two randomly selected plants from each plot were sampled; depending on their developmental stage, the samples were then separated into leaves, stems, flowers and bolls. The LAI was determined by a scanner (Phantom 9800xl, MicroTek, Shanghai, China) and ImagePro Plus (Media Cybernetics, Inc.), after which the leaves, stems and bolls were oven dried at 80°C to constant weight to determine their biomass.

#### 2.4.3. Yield and yield components

At the beginning of October, the seed cotton was hand harvested three times per plot. The lint yield at three different times as well as boll cracking were determined after ginning. Yield components, boll weight and lint percentage (lint weight/seed cotton weight; ginned with a laboratory gin [MPSY-100A]), were determined by randomly harvesting 50 open bolls, drying them, and then weighing them during each harvest. For the total number of bolls, opening and green bolls in 2 m of each plot were harvested and the number of plants in these 2 m was recorded, from which the boll number per plant could be given. Multiplying boll number per plant and plant number per plot then divided by the area of plot would be the total number of bolls per area.

### 2.5. Statistical analysis

All the data were analyzed as a randomized complete block design by ANOVA; planting patterns were considered independent variables. PROC GLM (Ver. 9.2, SAS institute, Ltd., USA) and Duncan’s multiple range test at a significance level of 0.05 were used.

A polynomial correlation was fitted for light interception in terms of DAS. To simulate the changes in the fraction of squares, fraction of bolls and shedding rate, Surfer 13 was used. Line scatter was used to study the variation in light interception and LAI in terms of DAS. The cotton biomass accumulation in terms of DAS was fitted to a nonlinear model that exhibited a sigmoidal logistic function (type I) via Origin Pro 9 (Origin Lab), and Adobe Illustrator CS5 (Adobe) was used for plotting graphs.

## 3. Results

### 3.1. Light interception

**Fig 2:**
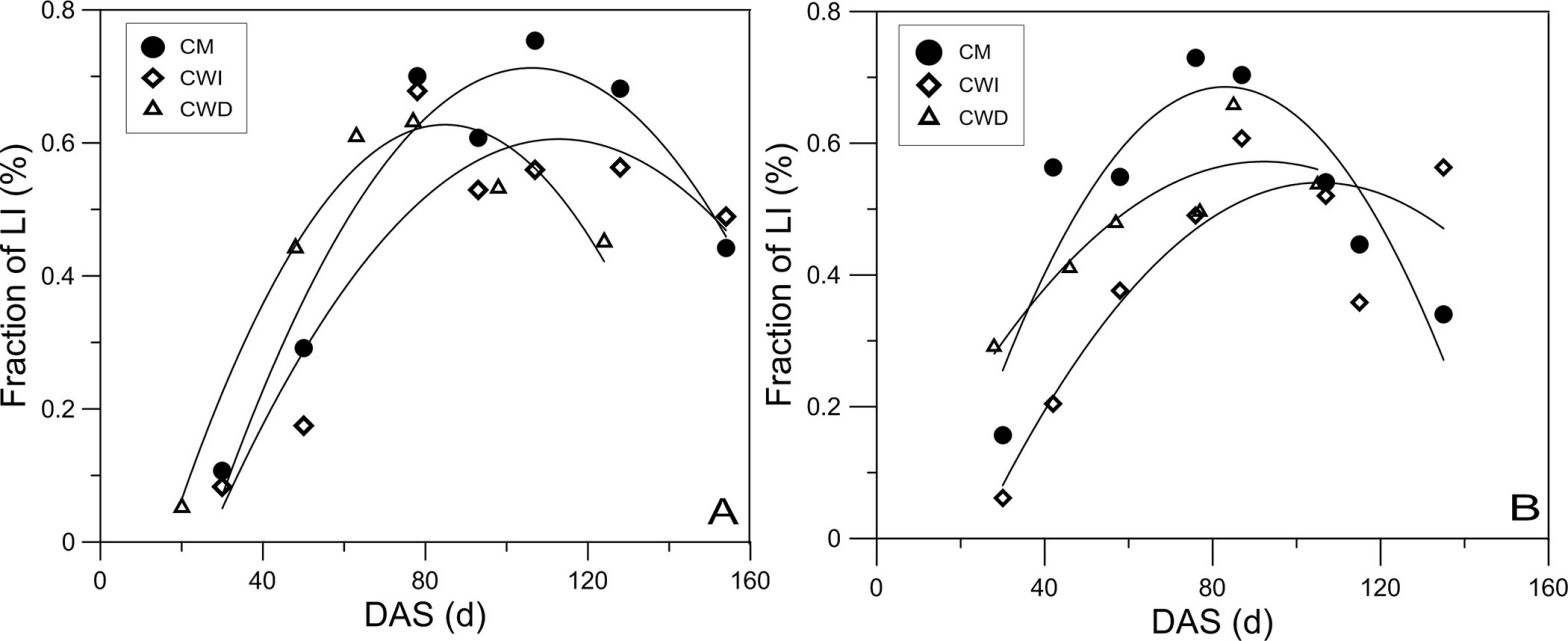
Fraction of light interception in the different planting patterns in 2016 (A) and 2017 (B).

In both years, the light interception in the CM, CWI and CWD increased in the early stage but then decreased (Fig 2). Owing to the short growth duration attributed to 30 days of late sowing, the light interception in the CWD both increased and decreased earlier than that in the CM and CWI. In both years, more light was captured in the CM than in the CWI throughout the cotton growth stages, except on the last sampling date. In addition, more light was intercepted in the CWD than in the CWI in the early cotton growth stage, which was attributed to the shedding effects of the wheat rows.

In 2016, the light interception in the CM was 22.4% and 51.4% higher than that in the CWI and CWD, respectively, at 30 DAS (Fig 2). At 107 DAS, both the CM and CWI presented their highest light interception, and 25.7% more light was captured in the CM than in the CWI. In 2017, the increase in light interception in the CM occurred from 30 DAS to 76 DAS, while in the CWI, this increase occurred from 30 to 87 DAS (Fig 2). Moreover, the light interception in the CM was 6.1% greater but 46.0% less than that in the CWI and CWD, respectively, at 30 DAS.

### 3.2. Fruit accumulation and shedding rate

#### 3.2.1. Square accumulation

**Fig 3:**
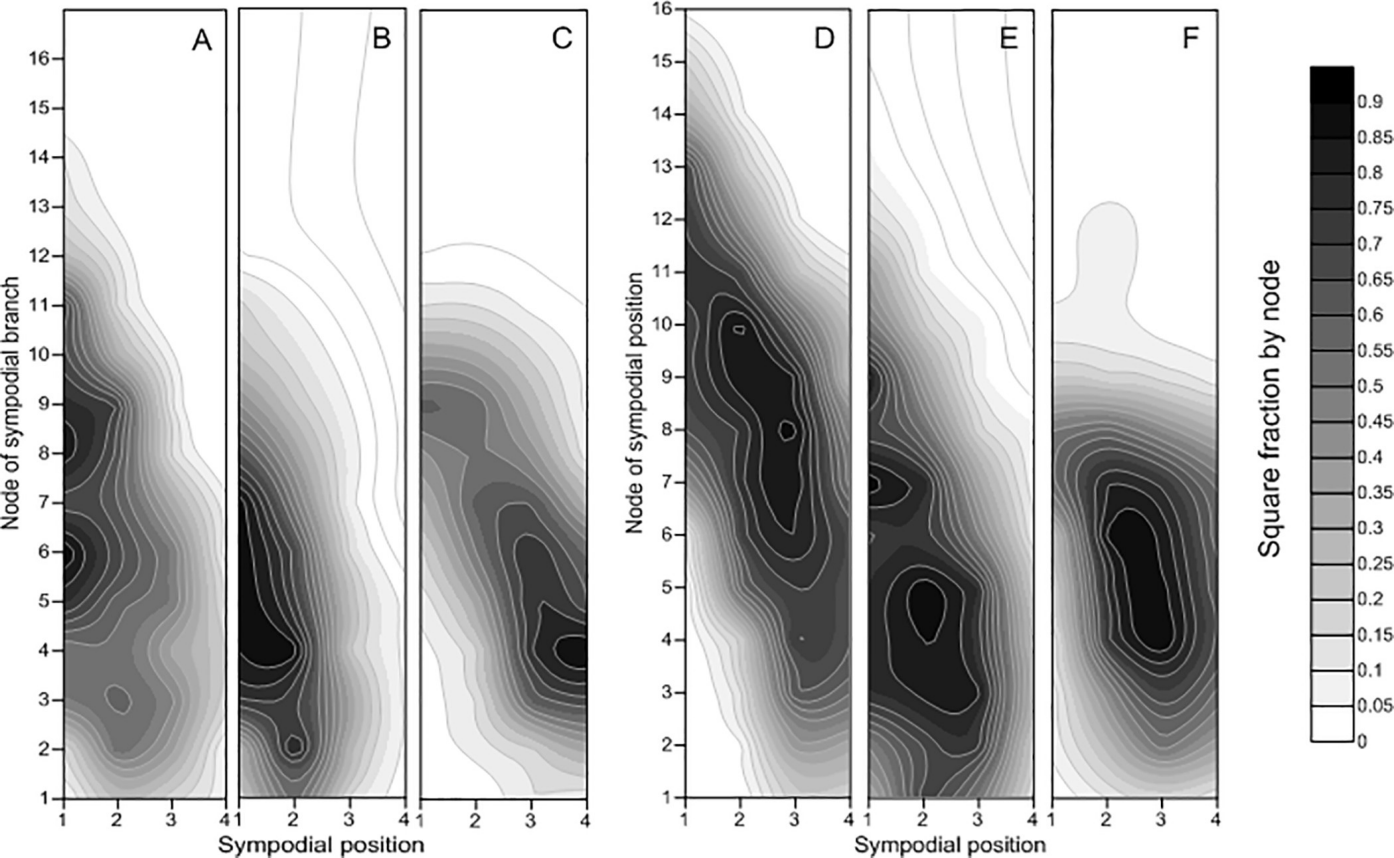
Fraction of squares of the different planting patterns at 75 DAS in 2016 and 2017. Note: A, B, and C represent the cotton monoculture (CM) system, the cotton/wheat intercropping (CWI) system, and the system in which cotton was directly seeded after wheat (CWD), respectively, in 2016; D, E, and F represent the cotton monoculture (CM) system, the cotton/wheat intercropping (CWI) system, and the system in which cotton was directly seeded after wheat (CWD), respectively, 2017.

On the one hand, the fraction of squares varied among the CM, CWI and CWD in both years of study (Fig 3). Compared with the CWI and CWD, the CM produced more squares on the upper nodes. However, the plants within the CM also presented differences among sympodial positions (SPs) and among nodes of fruit branches (FBs), showing that more squares were obtained on the lower FBs in both years. On the 9th, 10th and 11th FBs, the fraction of squares was lower in the CWI and CWD than in the CM.

#### 3.2.2. Boll accumulation

**Fig 4:**
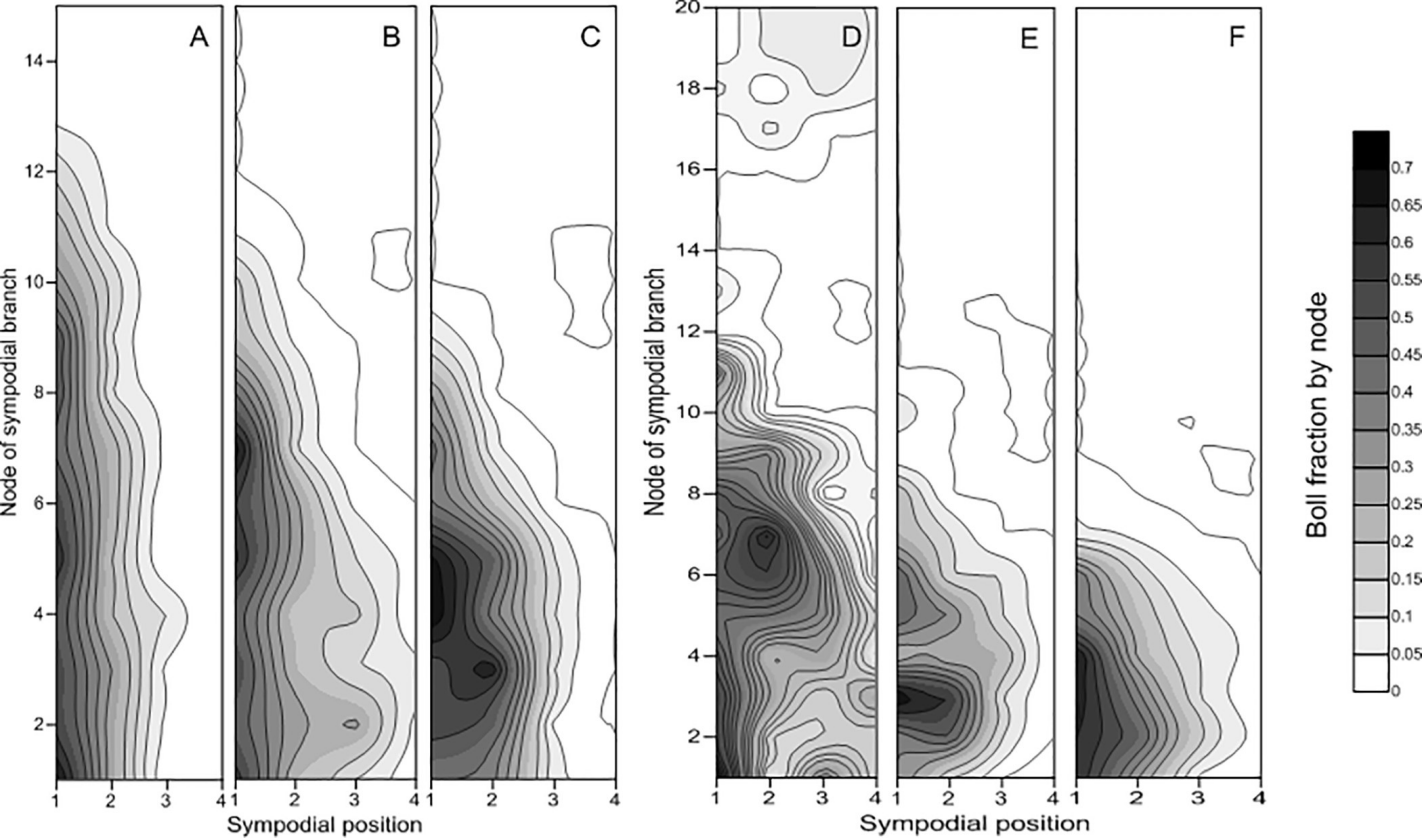
Fraction of bolls of the different planting patterns at 100 DAS in 2016 and 2017. Note: A, B, and C represent the cotton monoculture (CM) system, the cotton/wheat intercropping (CWI) system, and the system in which cotton was directly seeded after wheat (CWD), respectively, in 2016; D, E, and F represent the cotton monoculture (CM) system, the cotton/wheat intercropping (CWI) system, and the system in which cotton was directly seeded after wheat (CWD), respectively, in 2017.

Differences in boll distribution at 100 DAS are shown in Fig 4. The plants in both seasons were characterized not only by efficient boll retention close to the center nodes but also by very limited numbers of bolls on the upper nodes (Fig 4). Moreover, compared with the CWI and CWD, the CM resulted in the production of more bolls on the upper nodes in both years of the study. Additionally, SP 1 produced maximum bolls for all nodes. More bolls occurred at SP 3 and SP 4 in the CWI and CWD than in the CM in 2016.

#### 3.2.3. Shedding rate

**Fig 5:**
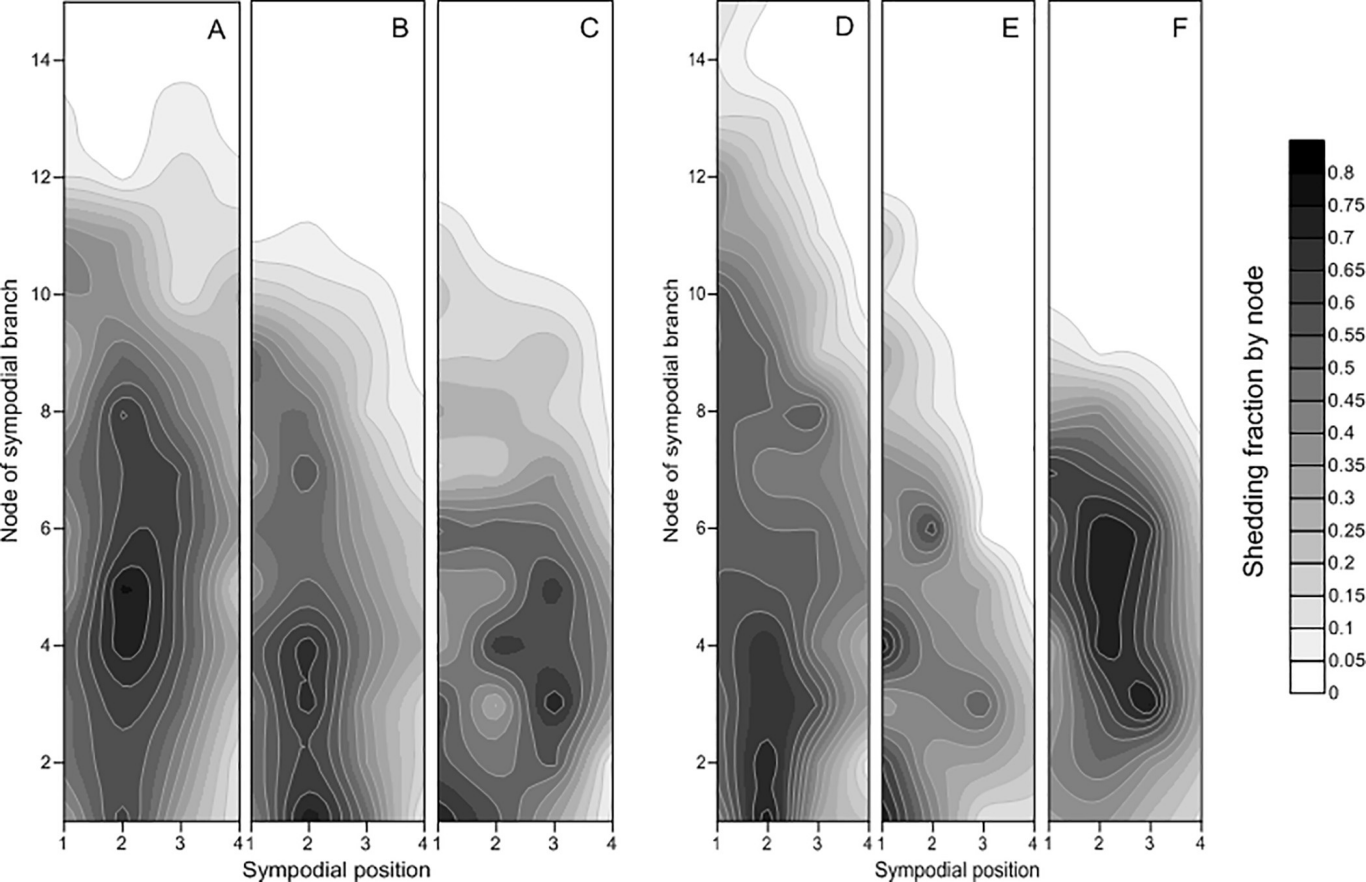
Shedding rate by node in the different planting patterns at 100 DAS in 2016 and 2017. Note: A, B, and C represent the cotton monoculture (CM) system, the cotton/wheat intercropping (CWI) system, and the system in which cotton was directly seeded after wheat (CWD), respectively, in 2016; D, E, and F represent the cotton monoculture (CM) system, the cotton/wheat intercropping (CWI) system, and the system in which cotton was directly seeded after wheat (CWD), respectively, in 2017.

The shedding rate on SP 1 was between 0 and 0.60 in 2016 and between 0 and 0.70 in 2017 (Fig 5). In 2016, the highest shedding rates in the CM (0.50), CWI (0.57) and CWD (0.60) all occurred on SP 1 of FB 4 and were 14% and 20% lower in the CM plots than in the CWI and CWD plots, respectively. In 2017, the highest shedding rate (0.65) in the CM occurred on SP 1 of FB 10, but in the CWI and CWD, the highest rates were observed on SP 1 of FB 4 and FB 7, respectively.

### 3.3. Cotton plant growth characteristics

#### 3.3.1. Differences in the LAI

**Fig 6:**
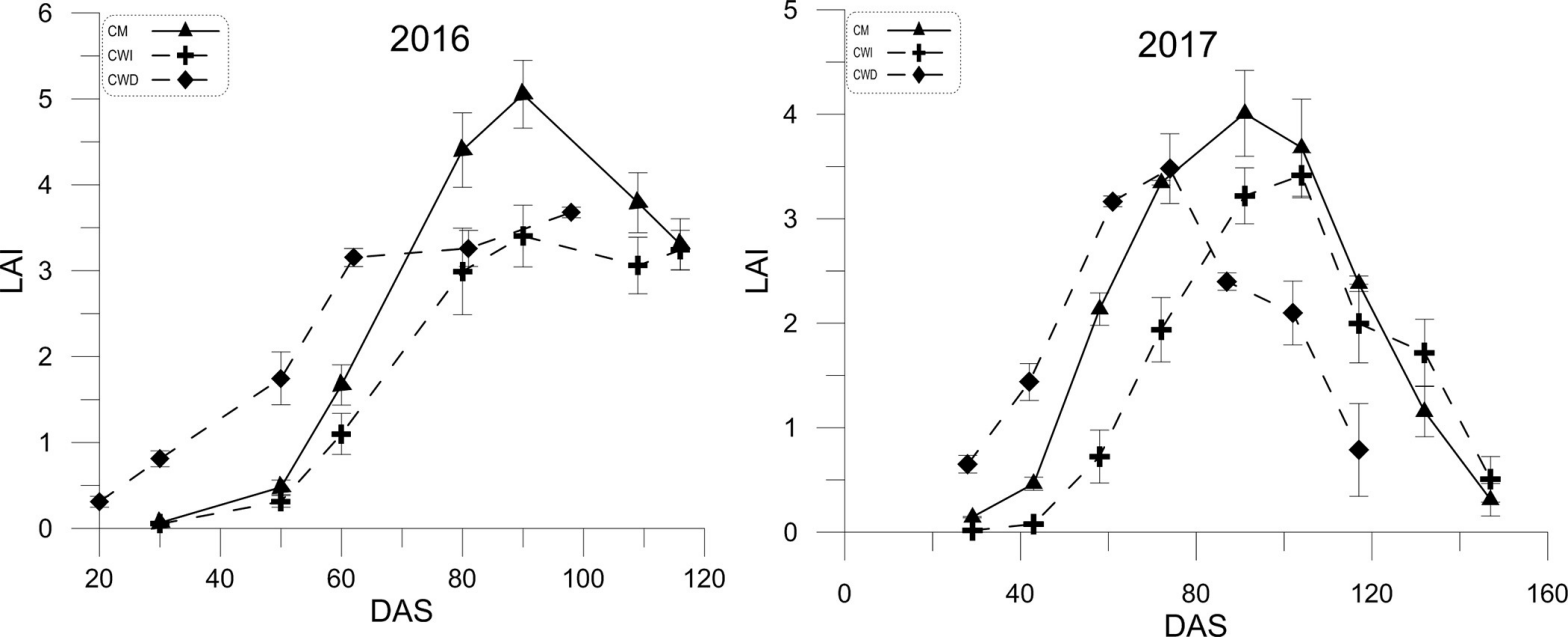
The LAI in terms of DAS of the different planting patterns in 2016 and 2017. Note: The error bars represent the standard deviations (STDEVs) (n = 3).

Among all the treatments, significant differences in the LAI were observed (Fig 6). The peak LAI occurred slightly later in the CWI than in the CM in both years; however, the cotton plants in the CWD provided more groundcover than did those in the CWI during the early growth stage in both years (Fig 6). The CM had a higher LAI than did CWI, except at the last two sampling times.

#### 3.3.2. Variation in biomass accumulation

**Fig 7:**
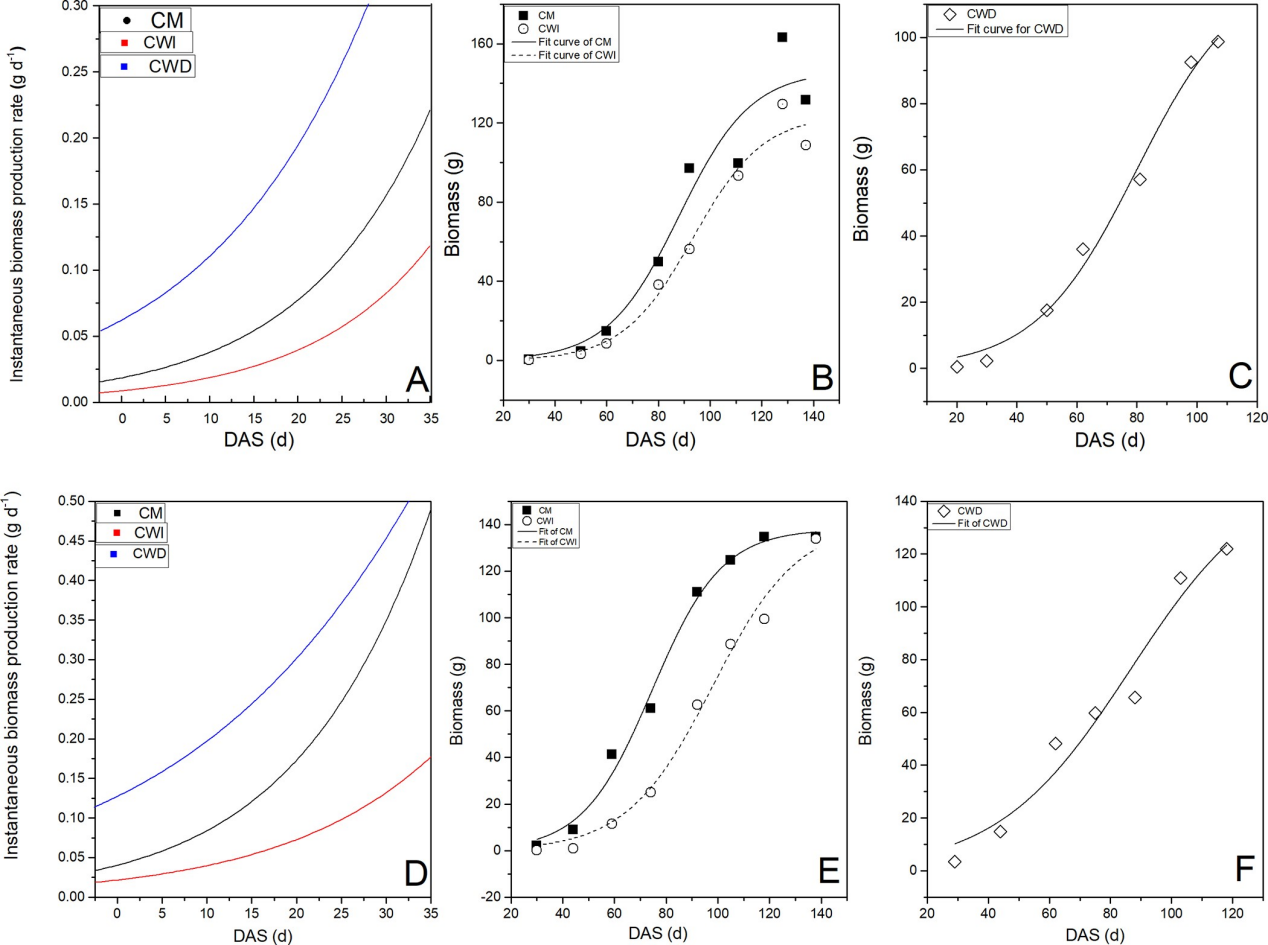
Sigmoidal fit of biomass accumulation and instantaneous biomass production rate for the different planting patterns in 2016 and 2017. Note: A, B and C represent the results of 2016; D, E, and F represent the results of 2017.

The biomass accumulation of the cotton plants was significantly influenced by planting patterns (Fig 7). The biomass exhibited a normal sigmoidal growth curve; the accumulation began at 50 DAS and ended at 110 DAS in the CM and CWI, yet a constant increase started from 40 DAS in the CWD. The instantaneous biomass production rate showed that the greatest daily biomass production was obtained in the CWD, followed by the CM and CWI, in 2016. At 92 DAS, the biomass in the CM was 42.1% higher than that in the CWI but only 4.6% higher than that in the CWD. Furthermore, compared with that in the CWI and CWD, the biomass in the CM increased by 6.1% and 0.8%, respectively, at 110 DAS.

### 3.4. Yield and yield components

The cotton yield and yield components were significantly influenced by planting patterns (Table 3). Compared with the CWI and CWD, the CM produced 12.6% and 36.1% greater seed cotton yield, respectively, in 2016. In 2017, 24.4% and 48.7% more seed cotton yield was obtained in the CM than in the CWI and CWD, respectively. With respect to lint yield, no significant differences were observed between the CM and CWI in 2016; in contrast, in 2017, the lint yield was 27.9% greater in the CM than in the CWI.

**Table 3:** Yield and yield components of different planting systems in 2016 and 2017.

Treatment	Seed cotton yield		Lint yield		Number of bolls		Boll weight		Lint percentage		Flowers before frost	
	(kg ha^-1^)		(kg ha^-1^)		(10^4^ ha^-1^)		(g)		(%)		(%)	
	2016	2017	2016	2017	2016	2017	2016	2017	2016	2017	2016	2017
CM	3757.76 a	4216.03 a	1419.69 a	1659.25 a	96.81 a	82.11 a	4.93 a	5.12 a	39.26 a	39.46 a	84.58 a	n.d.
CWI	3284.98 a	3186.68 b	1276.97 a	1196.36 b	78.50 b	71.20 ab	4.88 a	4.77 b	38.53 a	38.34 a	72.65 a	
CWD	2402.83 b	2160.79 c	814.26 b	784.19 c	68.42 b	60.50 b	4.55 a	4.32 c	38.48 a	36.64 b	14.10 b	

Note: CM, CWI, and CWD represent a cotton monoculture system, a cotton/wheat intercropping system, and a system in which cotton was directly seeded after wheat, respectively. The same letter within the same column subdivision means no significant differences according to LSD_0.05_. n.d. = not determined.

In terms of yield components, compared with that in the CWI and CWD, the boll number in the CM significantly increased, while no significant differences in boll numbers were observed between the CWI and CWD. In 2016, there were no significant differences in boll weight and lint percentage among treatments; however, the boll weight in the CM was 6.8% and 15.6% greater than that in the CWI and CWD, respectively. Before the frost, flower maturity did not significantly differ between the CM and CWI, but an 83.3% decrease occurred in the CWD.

## 4. Discussion

### 4.1. Variation of light interception in different cropping systems

The previous study found that the high productivity of intercrops, compared to monocultures, for the reason of an increase in accumulated light interception per unit cultivated area [17], but in this study we only compared light interception by cotton because it is important to improve light interception by the second crops, as there is no other crops influencing the previous crops. While as the shaded effect of wheat in CWI inhabited the establish of cotton, so light interception in CWI was less than in CM at the early growth period, but finally reached and exceeded the interception value of CM. In terms of CWD, improved establish was obtained with more light interception at the seedling stage than in CM and CWI. To increase light interception in CWI and CWD, using early maturity cultivar might be favorable to increase harvestable bolls [18], because comparatively growth delay occurred in CWI and CWD.

### 4.2. Relationships between light interception, the LAI, biomass and yield

The results of previous studies concerning productivity and resource use in cotton/wheat intercropping systems showed that cotton lint yield was significantly lower in intercropped systems than in monoculture systems, which is consistent with the results of the present study [19]. On one hand, compared with the CM, the CWI decreased cotton yields because of resource competition, and these decreases were associated with decreased boll numbers due to delayed fruit formation and reduced sink capacity [4, 9]. For instance, lint yield was greater in the CM than in the CWI and CWD; this greater yield was associated with 18.9% more bolls in 2016 and 6.8% increased boll weight in 2017. On the other hand, these increases in seed cotton and lint yield were associated with a longer growth duration in the CM than in the CWD and the avoidance of shading effects of wheat rows in the CWI.

In addition, cotton yield is determined by biomass accumulation and its partitioned proportions to reproductive organs [11]. Nevertheless, in recent years, cotton plants in CWD systems have suffered from the delayed planting of succeeding crops, which results in tremendous yield losses of the succeeding crops [7]. However, no significant negative effects of late sowing on the succeeding cotton canopy light capture capacity, LAI or instantaneous biomass production rate were observed in either season in the present study. Significant differences in the instantaneous biomass production rate among the treatments indicated that the initial exponential growth rate was greatest in the CWD.

Furthermore, biomass was strongly determined by resource capture [12]. Efforts have been made to increase light interception to maintain biomass accumulation and yield [20]. Here, the effects of planting patterns on light interception were studied, and the results suggested that, by capturing more light, this approach is an economical and feasible method for increasing cotton yields [21]. Therefore, increased biomass in the CM was attributed primarily to greater light interception than that in the CWI and CWD, which in turn promoted square formation and translocation to the developing bolls. Moreover, the greater light interception increased the fruit retention capacity; in contrast, poor light penetration into the canopy in the CWI led to the lowest LAI at the cotton seedling stage, which resulted in a reduction in light interception. The higher LAI in the CM and CWD than in the CWI during the early and middle growth stages promoted both canopy photosynthetic capacity and assimilate translocation toward the developing fruits. Hence, either reduced biomass accumulation or partitioning to reproductive growth reduced the cotton yield in the CWI compared with the CM.

### 4.3. Fruit distribution and shedding rate

As interspecific competition usually reduces the survival, growth or reproduction of at least one species [21], in the present study, more squares were found on the upper nodes of cotton plants in the CM than on those in the CWI and CWD, indicating that the CM presented the most compact fruiting habits. Moreover, on the basis of the findings that increased squares occurred on the upper nodes and outer positions, the growth rate of cotton plants in the CM and CWD was faster than that in the CWI at the squaring stage. Additionally, fewer squares were observed on the outer positions in the CWI than in the CM, which represented more rapid square development in the CM plots than in the other plots [19].

As bolls produced on the first positions of sympodial branches have been linked to increased maturity and cotton yield has been positively related to fruit maturity and harvestable boll numbers [8], the greatest fraction of bolls on the first positions in the CM plots strongly explained the greater seed cotton yield obtained in the CM compared with the CWI and CWD. High temperatures at the seedling stage and low temperatures at harvest in the CWD improved the early development of cotton plants but slowed the process of boll development, resulting in a shorter cotton seedling stage and effective boll development stage, which then dramatically reduced the lint yield of those plots compared with the CM plots. The cotton plants in the CWI exhibited a pronounced delay due to initial shading from the wheat rows at the cotton seedling stage. These cotton plants endured the longest seedling stage and consequently exhibited delayed reproductive growth because of the interaction between the two component crops before wheat harvest [22]. Therefore, the cotton plants in the CWI exhibited a delay in square formation, contributing to decreased numbers of bolls and less lint yield. Owing to their delayed sowing, the cotton plants in the CWD were easily affected by the relatively low temperatures during the flowering and boll-formation periods. These phenomena consequently delayed reproductive development, which affected cotton biomass accumulation and distribution and ultimately limited crop productivity.

## 5. Conclusions

In modern agricultural systems, management concerning planting patterns is one method to increase cotton yields, especially given the sharp reduction in cotton planting area in the Yellow River Valley of China. This study found the greatest light capture ability in cotton monoculture system from 80 to 120 DAS. While for the other two intercropping systems, cotton directly sown after wheat system exhibited higher light interception than cotton/wheat relay intercropping system. Comparing with cotton/wheat relay intercropping system and cotton seeded directly after wheat system, the greatest fraction of bolls on the first positions was produced in the cotton monoculture plots, which strongly explained the greater seed cotton yield obtained in the monocultured system. Even though the early development of cotton plants in cotton seeded directly after wheat cropping system was much better improved than plants in the other two cropping systems, but challenged with shortening boll developing duration, therefore the lint yield decreased.
